# Oligomeric amyloid-beta induces MAPK-mediated activation of brain cytosolic and calcium-independent phospholipase A_2_ in a spatial-specific manner

**DOI:** 10.1186/s40478-017-0460-6

**Published:** 2017-07-27

**Authors:** Juan Pablo Palavicini, Chunyan Wang, Linyuan Chen, Kristen Hosang, Jianing Wang, Takami Tomiyama, Hiroshi Mori, Xianlin Han

**Affiliations:** 1 Center for Metabolic Origins of Disease, Sanford Burnham Prebys Medical Discovery Institute, 6400 Sanger Road, Orlando, FL 32827 USA; 2 Department of Translational Neuroscience, Osaka City University Graduate School of Medicine, 1-4-3 Asahimachi, Abeno-ku, Osaka, 545-8585 Japan; 3 Department of Clinical Neuroscience, Osaka City University Graduate School of Medicine, 1-4-3 Asahimachi, Abeno-ku, Osaka, 545-8585 Japan

**Keywords:** Alzheimer’s disease, Amyloid-beta, Fatty acid, Lysophospholipid, Phospholipase A_2_, Oxidative stress

## Abstract

Alzheimer’s disease (AD) is histopathologically characterized by the build-up of fibrillar amyloid beta (Aβ) in the form of amyloid plaques and the development of intraneuronal neurofibrillary tangles consisting of aggregated hyperphosphorylated Tau. Although amyloid fibrils were originally considered responsible for AD pathogenesis, recent convincing evidence strongly implicates soluble oligomeric Aβ as the primary neurotoxic species driving disease progression. A third largely ignored pathological hallmark, originally described by Alois Alzheimer, is the presence of “adipose inclusions”, suggestive of aberrant lipid metabolism. The molecular mechanisms underlying these “lipoid granules”, as well as their potential link to soluble and/or fibrillar Aβ remain largely unknown. Seeking to better-understand these conundrums, we took advantage of the powerful technology of multidimensional mass spectrometry-based shotgun lipidomics and an AD transgenic mouse model overexpressing mutant amyloid precursor protein (APP E693Δ-Osaka-), where AD-like pathology and neurodegeneration occur as a consequence of oligomeric Aβ accumulation in the absence of amyloid plaques. Our results revealed for the first time that APP overexpression and oligomeric Aβ accumulation lead to an additive global accumulation of nonesterified polyunsaturated fatty acids (PUFAs) independently of amyloid plaques. Furthermore, we revealed that this accumulation is mediated by an increase in phospholipase A_2_ (PLA_2_) activity, evidenced by an accumulation of *sn*-1 lysophosphatidylcholine and by MAPK-mediated phosphorylation/activation of group IV Ca^2+^-dependent cytosolic (cPLA_2_) and the group VI Ca^2+^-independent PLA_2_ (iPLA_2_) independently of PKC. We further revealed that Aβ-induced oxidative stress also disrupts lipid metabolism via reactive oxygen species-mediated phospholipid cleavage leading to increased *sn*-2 lysophosphatidylcholine as well as lipid peroxidation and the subsequent accumulation of 4-hydroxynonenal. Brain histological studies implicated cPLA_2_ activity with arachidonic acid accumulation within myelin-rich regions, and iPLA_2_ activity with docosahexaenoic acid accumulation within pyramidal neuron-rich regions. Taken together, our results suggest that PLA_2_-mediated accumulation of free PUFAs drives AD-related disruption of brain lipid metabolism.

## Introduction

Decades of Alzheimer’s disease (AD) research have been grounded on the so called “amyloid cascade hypothesis”, which originally placed amyloid precursor protein (APP) mismetabolism and subsequent Aβ aggregation (i.e., fibrillation) as the initial trigger responsible for instigating further pathological events (i.e., tauopathy, synaptic damage, and neuronal death) [49, 52, 97]. However, amyloid deposits were later shown to correlate poorly with cognitive decline and to be disconnected from Aβ-induced toxicity [29, 68, 72, 85]. On the other hand, characterization of soluble Aβ structures led to the discovery of Aβ derived diffusible ligands (ADDLs) or oligomeric Aβ [63]: extremely neurotoxic species that strongly correlate with synaptic impairment and parallel cognitive decline in animal models and humans [11, 36, 53, 62, 63, 68, 120, 122, 123]. Importantly, it has been shown that oligomeric Aβ species are both necessary and sufficient to disrupt cognitive function in vivo [21, 64, 107]. These findings led to a “revised” amyloid cascade hypothesis where diffusible oligomeric Aβ replaced fibrillar Aβ as the central neurotoxic event driving AD pathogenesis [48, 98].

The E693Δ (Osaka) mutation in APP, which was found in Japanese pedigrees, causes familial AD by enhancing Aβ oligomerization in the absence of deposits of amyloid plaques [116]. The mutant Aβ peptide, which lacks glutamate-22 (E22Δ), forms abundant oligomers in vitro and causes endoplasmic reticulum stress-induced apoptosis in cultured cells [83]. When injected into rat cerebral ventricle, synthetic mutant Aβ E22Δ peptide inhibits hippocampal long-term potentiation more potently than wild-type (WT) peptide [116]. Exogenously applied Aβ E22Δ peptide induces dose-dependent loss of synapses in mouse hippocampal slices [112]. In addition, APP_E693Δ_ transgenic mice (APP_OSK_) showed intraneuronal accumulation of Aβ oligomers, synapse loss, memory impairment, and significant neuronal loss at 24 months of age [115]. Thus, APP_OSK_ mice successfully recapitulate Aβ neurotoxicity in the absence of amyloid plaques.

When Alois Alzheimer first described the disease over 100 years ago, he identified abnormal protein deposits as well as adipose saccules (lipid inclusions) in the brains of his patients [4]. These observations suggested a possible relation between AD and lipid imbalance, which was established decades later when the strongest genetic risk factor in AD was linked to apolipoprotein E (apoE), the major lipid transporter in the CNS. Dysregulation of multiple lipid families has been linked to AD, including alterations in the levels of sulfatide, plasmalogen ethanolamine glycerophospholipid, cholesterol, ceramide, and fatty acids (FAs) [15, 18, 19, 23, 26, 41, 43, 44, 46].

FAs and their metabolites are of particular relevance, given that they participate in processes involved in the pathogenesis of AD, including synaptic plasticity, inflammation, cerebrovascular function, and oxidative stress [74, 88, 89, 106]. FAs are released from phospholipids by phospholipase A_2_ (PLA_2_) [61], a family of enzymes that catalyze the cleavage of FAs from the *sn*-2 position of phospholipids. These enzymes are not only important for maintenance of cellular membrane phospholipids, they also play a key role in regulating the release of signaling molecules like arachidonic acid (AA) and docosahexaenoic acid (DHA), important precursors for lipid-derived modulators of cell signaling and inflammatory processes. Given that phospholipids within CNS membranes are enriched in polyunsaturated fatty acids (PUFAs) [110] and that the *sn*-2 position is mostly constituted with unsaturated FAs, PLA_2_ cleavage activity within the brain results in accumulation of lysophospholipids and unsaturated FAs [93, 96, 134].

In the mammalian system, more than 19 different isoforms of PLA_2_ have been identified, and different PLA_2_s have been shown to participate in physiological events related to cell injury, inflammation, and apoptosis [24, 81]. Research to understand PLA_2_s in the CNS has focused on 3 PLA_2_ isoforms: the group IV Ca^2+^-dependent cytosolic PLA_2_ (cPLA_2_), which has been strongly associated with AD (reviewed in [94]) [22, 95, 108, 109, 111, 113]; the group VI Ca^2+^-independent PLA_2_ (iPLA_2_), which has been proposed to account for >70% of brain PLA_2_ activity [132] and is highly enriched in AD-affected brain regions (i.e., cortex and hippocampus) [87]; and the group II secretory PLA_2_ (sPLA_2_), which has also been linked to AD more recently [14, 80].

Importantly, the activity of cPLA_2_ has been shown to be tightly regulated by multiple mechanisms. First, cPLA_2_ becomes activated after translocating to the plasma membrane from the cytosol [35]. Although Ca^2+^ is not necessary for cPLA_2_ catalytic activity, nanomolar Ca^2+^ concentrations are needed for its binding to the membrane [32]. Second, it is well-established that phosphorylation of cPLA_2_α at multiple sites (Ser505 and Ser515) stimulates its catalytic activity [30, 34, 50, 60, 66]. In vitro work has revealed that protein kinase C (PKC) plays an important role in mediating cPLA_2_ phosphorylation and AA release in murine astrocytes through both MAPK-dependent and MAPK-independent pathways [131]. Third, cPLA_2_ regulation via protease-mediated cleavage has also been documented [1, 5, 6, 30, 40, 119, 128], although this regulation seems to occur only under apoptotic and/or necrotic conditions. Conflicting data have been reported regarding the effects of cPLA_2_ proteolysis which has been found to both activate [30, 40, 128] and inhibit [1, 5, 6, 119] its activity. Notably, cPLA is highly specific to AA cleavage/release [28, 100, 101] and to PC [81]. In fact, cPLA_2_α-deficient mice fail to generate AA metabolites after brain injury [9, 61], thus cPLA_2_α seems to be the most relevant cPLA_2_ in the brain.

Like cPLA_2_, the activity of iPLA_2_ also seems to be tightly controlled. First, it has been well-established that iPLA_2_ is inhibited by calmodulin and activated by Ca^2+^ release from ER where calcium influx factor (CIF) has been proposed to displace inhibitory calmodulin [104, 129, 130]. Second, it has been proposed that PKC mediates phosphorylation of iPLA_2_ (directly and/or indirectly) promoting its activity [75]. Third, caspase-3-dependent cleavage and activation of iPLA_2_ have been documented [136]. Notably, murine studies have reported expression of an 80-kDa iPLA_2_ isoform (iPLA_2_β encoded by the *PLA2G6* gene) in brain tissue [132]. iPLA_2_β has been shown to be physiologically and clinically relevant, as demonstrated by characterization of iPLA_2_β-KO mice which model neurodegeneration with brain iron accumulation [70, 102] and by the fact that mutations in the *PLA2G6* gene lead to two childhood neurologic disorders [39, 56, 78].

Although multiple lipid classes and lipid cleavage enzymes have been associated to AD, whether lipid dysregulation plays a causative or epiphenomal role in the disease remains largely unknown. In the current study, we took advantage of the APP_OSK_ mouse model where AD-like pathology and neurodegeneration occur in the absence of amyloid plaques, and demonstrated that oligomeric amyloid-beta (Aβ) induces accumulation of free PUFAs and lysophosphatidylcholine by activation of brain cPLA_2_ and iPLA_2_ within myelin-rich and pyramidal neuron-rich regions, respectively, via MAPK-mediated phosphorylation in a PKC-independent manner.

## Materials and methods

### Mice

Brain tissue from 12 and 24 month old APP_OSK_-Tg, APP_WT_-Tg, and non-Tg mice (*n* = 4/genotype including an equal mix of male and female mice) was kindly obtained from Dr. Takami Tomiyama, Associate Professor from the Osaka City University. As previously described, three lines of APP-Tg lines have been established for APP_WT_ and APP_OSK_ mice with high (L1), low (L2), and intermediate (L3) human expression of the transgene [115]. All the studies were performed using L1 APP-Tg lines. It is important to note that APP_WT_ L1 mice express higher levels of human APP (2-fold) than APP_OSK_ L1 mice do [115].

### Lipid extraction

Mouse cerebrum tissue was sub-dissected by removal of olfactory lobe, cerebellum, brain stem, and colliculus from each hemibrain. Frozen cerebrum samples were weighed, lyophilized, pulverized, and homogenized in 500 μl of ice-cold diluted phosphate-buffered saline (0.1X PBS) on a cooling tissue homogenizer (Cryolys Precellys Evolution Homogenizer). Protein assays on individual homogenates were performed using a BCA protein assay kit (Pierce, Rockford, IL, USA). Lipids were extracted by a modified procedure of Bligh and Dyer extraction as described previously [16, 17] in the presence of internal standards which were added based on total protein content of the sample.

### Mass spectrometric analysis of lipids

A triple-quadrupole mass spectrometer (Thermo Scientific TSQ Vantage, CA, USA) equipped with a Nanomate device (Advion Bioscience Ltd., NY, USA) and Xcalibur system software was used as previously described [47, 133]. Diluted lipid extracts were directly infused into the ESI source through a Nanomate device [47]. Typically, signals were averaged over a 1-min period in the profile mode for each full scan MS spectrum. For tandem MS, a collision gas pressure was set at 1.0 mTorr, but the collision energy varied with the classes of lipids as described previously [45, 133]. Similarly, a 2- to 5-min period of signal averaging in the profile mode was employed for each tandem MS mass spectrum. All full and tandem MS mass spectra were automatically acquired using a customized sequence subroutine operated under Xcalibur software. Data processing including ion peak selection, baseline correction, data transfer, peak intensity comparison, ^13^C deisotoping, and quantitation were conducted using a custom programmed Microsoft Excel macro as previously described [133] after considering the principles of lipidomics [125].

### Elisa

The levels of Aβ oligomers were quantified by direct ELISA with anti-human amyloid-β E22P (11A1) mouse IgG monoclonal antibody (IBL, Japan) at 1 μg/ml as previously described [13, 118]. Briefly, PBS supernatants (at a concentration of 5000 μg/ml of total protein) were diluted 6-fold in sodium bicarbonate pH 9.6 (0.5X ELISA Plate Coating buffer, Alpha Diagnostic International, TX) and allowed to coat ELISA plates at 50 μl/well. After incubation with HRP-conjugated anti-mouse IgG, 11A1 immunoreactivity was detected using 3,3′,5,5′-Tetramethylbenzidine (TMB-1, Alpha Diagnostic International, TX). Reactions were stopped with diluted sulfuric acid (1X Stop Solution, Alpha Diagnostic International, TX).

### Western blot analysis

Pulverized cerebrum tissues were homogenized in 1X NP40 on a cooling tissue homogenizer (Cryolys Precellys Evolution Homogenizer). NP40 homogenates were centrifuged at 12,300 rpm for 20 min at 4 °C and supernatants were run into NuPage 4–12% Bis-Tris (Life Technologies, NY) under reducing conditions. Samples were normalized based on total protein content, which was estimated by the BCA protein assay. Western blot analyses were performed using antibodies against cPLA_2_ (sc-454 and sc-376,636, Santa Cruz Biotechnology -SCB-), phospho-cPLA_2_ -S505- (2831, Cell Signaling Technology -CST-), iPLA_2_ (sc-376,563, SCB; and NBP1–81586, Novus), MAPK p42/p44 (4695, CST), phospho-MAPK p42/p44 -T202/Y204- (4370, CST), MAPK p38 (8690, CST), phospho-MAPK p38 (rabbit polyclonal, CST), SAPK/JNK (9252, CST), active JNK (V7931, Promega), PKCα (2056, CST), PKCδ (9616, CST), PKCλ (610,207, BD Biosciences), phospho-PKC pan -βII Ser660- (9371, CST), phospho-PKCα/βII -T638/641- (9375, CST), phosphor-PKCδ -T505- (9374, CST), phospho-PKCζ/λ -T410/403- (9378, CST), CaMK2 (sc9035, SCB), pCaMK2 (sc-12,886-R, SCB), β-Tubulin (2146, CST), GAPDH (MAB374, Millipore), VDAC (4866, CST). Relative intensities were quantified using ImageJ software.

### MALDI imaging

Matrix-assisted laser desorption/ionization (MALDI) imaging of fatty acids was carried out as previously described [124]. Briefly, fresh frozen brain from adult C57BL/6 J WT mouse was cryosectioned at 10-μm thickness. Brain slices were transferred onto the conductive side of indium tin oxide (ITO) slides and desiccated in vacuum for 30–60 min. After drying, N-(1-naphthyl) ethylenediamine dihydrochloride matrix was applied by the Bruker ImagePrep device (Bruker Daltonics, Bremen, Germany). MALDI mass spectra were acquired in the negative ion mode using a reflectron geometry MALDI-TOF mass spectrometer (Ultraflextreme; Bruker Daltonics) equipped with a neodymium-doped yttrium aluminum garnet (Nd:YAG)/355-nm laser as the excitation source. Imaging data were analyzed using FlexImaging v3.0 and BioMap v3.8. Ion images were generated with a bin width of ±0.2 Da. The normalization method was total ion count (TIC).

### Immunofluorescence

Mouse brains were dissected, fixed in 4% paraformaldehyde, cryoprotected, embedded in OCT, and frozen. Cryostat brain sections (8 μm) were mounted on positively charged slides. Tissue-containing slides were incubated with phospho-cPLA2 -S505- (2831, CST), iPLA2 (NBP1–81586, Novus), and NeuN (NAB377, Millipore) primary antibodies overnight at 4 °C and incubated with secondary antibody (Goat anti-mouse Alexa Fluor® 555, Goat anti-rabbit Alexa Fluor® 647) for 1 h at room temperature, followed by the addition of DAPI-containing mounting media (Vectashield, Vector Laboratories). Images were taken using 20× and 40× objectives on a Nikon A1R VAAS inverted confocal microscope and analyzed using NIS-Elements imaging software (Nikon).

### Statistical analysis

Quantitative data were normalized to protein content and were presented as the means ± SE. Differences between mean values were determined by unpaired Student’s *t* test (one time point analysis comparing the abundance of specific lipid species or protein between the 3 different genotypes) using GraphPad Prism software.

## Results

### APP overexpression and oligomeric Aβ accumulation lead to significant and additive increases in unsaturated nonesterified fatty acids

**Fig. 1:**
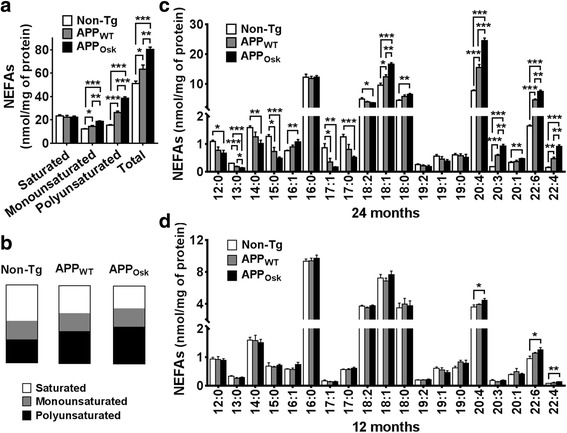
Effects of APP_WT_ and APP_OSK_ overexpression on the levels and proportions of nonesterified fatty acids in the brains of middle-age and old mice. Cerebrum samples (forebrain without olfactory lobe) from 12 and 24 month old non-Tg, APP_WT_, and APP_OSK_ were lyophilized, pulverized, and homogenized in PBS 0.1X buffer using a cooled bead beater followed by a modified Bligh and Dyer lipid extraction and AMPP derivatization. **a** Total masses of saturated, monounsaturated, polyunsaturated, and total nonesterified fatty acids (NEFAs) were quantified by MDMS-SL as described in “Materials and methods” and plotted using GraphPad Prism software. Data shown is from old mice (24 months of age). **b** Proportions of saturated, monounsaturated, and polyunsatured NEFAs for each genotype are shown as parts of whole graphs for old mice. Individual NEFA species with masses above 0.1 nmol/mg of protein were graphed for old (**c**) and middle-age (**d**) mice. The data represent means ± SE obtained from 4 animals/genotype. *p < 0.05, **p < 0.01, and ***p < 0.01

Analysis of nonesterified fatty acids (NEFAs, i.e., free FAs) within cerebrum (forebrain without olfactory lobe) homogenates from old (24-month-old) mice revealed that both APP overexpression (APP_WT_) and oligomeric Aβ accumulation (APP_OSK_) lead to significant and additive increases of total NEFAs (24% and 57% increase, respectively) compared to non-Tg controls (Fig. 1a). This increase occurred most dramatically within PUFAs, which increased 1.7-fold in APP_WT_ and 2.5-fold in APP_OSK_ mouse brains compared to non-Tg controls. Similarly, APP overexpression and high oligomeric Aβ content also led to a significant increase of monounsaturated FAs (MUFAs) (19% and 55% increase, respectively). On the other hand, the total content of saturated NEFAs was not affected either by APP overexpression or oligomeric Aβ accumulation (Fig. 1a). Consequentially, the proportion of PUFAs, which under physiological conditions (non-Tg) constitute about 30% of total NEFA content, increased to ~40% in APP_WT_ and to ~50% in APP_OSK_ (Fig. 1b). Conversely, the proportion of saturated NEFAs decreased from about 50% in non-Tg, to ~35% in APP_WT_ and to ~30% in APP_OSK_; meanwhile the proportion of MUFAs (~25%) remained unaltered between the 3 different genotypes (Fig. 1b).

Detailed characterization of NEFA molecular species of 24-month-old mice revealed that the most abundant NEFA under physiological conditions, palmitate (16:0), was not altered either by APP overexpression or oligomeric Aβ accumulation (Fig. 1c). On the other hand, under APP overexpression and oligomeric Aβ accumulation, AA (20:4) was markedly increased (2- and 3.2-fold in APP_WT_ and APP_OSK_, respectively), to such an extent that it became the most abundant NEFA in the brain. Importantly, altered AA metabolism has been implicated with neuroinflammation, neuronal death, and a number of neurological disorders [2, 3, 8, 31, 90, 114, 126]. Similarly, DHA (the second major PUFA in the brain) was also extensively (2.8-fold) and dramatically (4.5-fold) increased in APP_WT_ and APP_OSK_, respectively. It is important to note that DHA, like AA, can be metabolized by cyclooxygenase (COX) and lipoxygenase enzymes and generate active compounds [99]. DHA products, in contrast to AA products, seem to have a beneficial effect in inflammatory and neurodegenerative conditions. Thus, the increased levels of both AA and DHA in APP-Tg mice could lead to two opposite effects (pro- and anti-inflammatory, respectively). Less abundant long-chain PUFAs (like 22:4 or 20:3) were also substantially affected, showing 2- to 3-fold increases in APP_WT_ and 3- to 5-fold increases in APP_OSK_ compared to non-Tg controls. The most abundant MUFA, oleic acid (OA, 18:1), was also significantly increased (although less extensively) in both APP_WT_ and even further in APP_OSK_ mice. Less abundant MUFA species were either slightly increased only in APP_OSK_ mice (16:1 and 20:1), not significantly altered (19:1), or reduced (17:1) in APP-Tg mice. An additional interesting observation is that medium- and long-chain saturated NEFAs (12:0 to 15:0, and 17:0) were significantly decreased in both APP_WT_ and APP_OSK_ mice compared to controls (~30% and ~50% decrease, respectively), revealing that fatty acid metabolism undergoes significant remodeling under high APP and oligomeric Aβ content conditions (Fig. 1c).

Next, we proceeded to characterize the effects of APP_WT_ and APP_OSK_ overexpression on NEFA content in middle-aged mice (12-month-old). Although total NEFA content was not significantly altered within the brains of younger mice, the levels of the 2 major PUFAs (i.e., AA and DHA) were significantly increased in APP_OSK_ mice compared to non-Tg controls (~20% and ~30% increase, respectively); while saturated and monounsaturated NEFAs were not significantly altered in middle-aged mice (Fig. 1d).

**Fig. 2:**
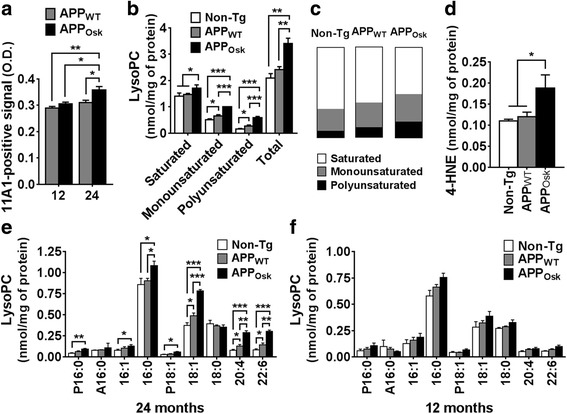
Age-dependent effects of oligomeric Aβ accumulation in APP_WT_ and APP_OSK_ mouse brains on the levels and proportions of lysophosphatidylcholines and 4-HNE. Cerebrum samples from 12 and 24 month old non-Tg, APP_WT_, and APP_OSK_ were lyophilized, pulverized, and homogenized in PBS 0.1X buffer using a cooled bead beater followed by a modified Bligh and Dyer lipid extraction. **a** PBS supernatants were subjected to direct oligomer Aβ -11A1- ELISA, O.D. values are shown. **b** Total masses of saturated, monounsaturated, polyunsaturated, and total lysophosphatidylcholines (lysoPCs) for old mice were quantified by MDMS-SL as described in “Materials and methods” and plotted using GraphPad Prism software. **c** Proportions of saturated, monounsaturated, and polyunsaturated lysoPCs for each genotype are shown as parts of whole graphs. **d** Total 4-HNE content in 24-month old mice. Individual lysoPC species with masses above 0.05 nmol/mg were graphed for old −24 months- (**e**) and middle-age − 12 months- mice (**f**). The data represent means ± SE obtained from 4 animals/genotype. *p < 0.05, **p < 0.01, and ***p < 0.01

As expected, ELISA analysis (using 11A1, an antibody against human oligomeric Aβ) revealed an age dependent increase of oligomeric Aβ content in APP_OSK_ mice, which accumulated higher levels of oligomeric Aβ than APP_WT_ controls (Fig. 2a). These results are consistent with previous reports, where high levels of human oligomeric Aβ have been reported in the brains of APP_OSK_ mice compared to APP_WT_ controls or non-Tg (which lack human Aβ) by Western blot, ELISA, and immunohistochemistry [115, 118].

### APP overexpression and oligomeric Aβ accumulation lead to a significant and additive increase of lysoPC content

The dramatic increases in long-chain PUFAs (particularly AA and DHA) on transgenic APP mice were strongly suggestive of increased PLA_2_ activity; thus, we proceeded to characterize the content of brain lysophospholipids (LPLs) in old and middle-age mice. As expected from the NEFA results, we observed an upward trend in total lysoPC in 24-month-old APP_WT_ mice (15%) and a significant increase in APP_OSK_ mice (63%) compared to non-Tg controls (Fig. 2b). PLA_2_ cleavage results in lysoPL molecular species containing a FA in the *sn*-1 position, which is typically occupied by saturated or MUFAs. Consistently, the levels of lysoPC containing a saturated FA increased in APP_OSK_ mice compared to APP_WT_ and non-Tg mice. Furthermore, the levels of lysoPC containing a MUFA were significantly and additively increased in APP_WT_ and APP_OSK_ (by 30% and 98%, respectively). Unexpectedly, the levels of lysoPC containing PUFAs were also significantly and additively increased in APP_WT_ and APP_OSK_ (1.7- and 3.7-fold increases, respectively, Fig. 2b).

The unexpected increase in PUFA-containing lysoPC in APP-Tg mice could theoretically be explained by (1) a higher proportion of PUFAs at the *sn*-1 position, (2) increased PLA_1_ activity, and/or (3) increased reactive oxygen species (ROS)-mediated lysoPC formation under pathological conditions (in APP-Tg mice). The fact that PUFA-containing lysoPCs constitute only a minority of the total lysoPCs (8%, 11%, and 18% in non-Tg, APP_WT_ and APP_OSK_, respectively, Fig. 2c) supports the notion that PLA_2_ activity in the brain is dominant over PLA_1_ under physio(patho)logical conditions and that PUFAs are preferentially located at the *sn*-2 position. Therefore, we propose that the increase in PUFA-containing lysoPCs is most likely due to high ROS production under high oxidative stress pathogenic conditions (APP_WT/OSK_ overexpression). Increased lysoPL, particularly those containing PUFAs at the *sn*-2 position resulting from plasmalogen (*sn*-1) phospholipid cleavage, is a well stablished oxidative stress signature [10, 33, 42, 51]. Consistent with our proposed model, we found that the levels of 4-hydroxynonenal (4-HNE), a product of oxidative stress induced lipid peroxidation, were significantly increased in APP_OSK_ mice compared to APP_WT_ and non-Tg controls (by 66%, *p* = 0.04) (Fig. 2d). In agreement with our data, increased ROS-generation has been reported in APP_OSK_ expressing COS-7 cells [84] and Aβ oligomers have been shown to induce neuronal oxidative stress [27].

Detailed characterization of lysoPC lipid species revealed that plasmalogen palmitic (P16:0), palmitoleic (16:1), and oleic acid (P18:1 and 18:1) containing lysoPC species were significantly and similarly increased (by 1.7- to 2.2-fold) in old APP_OSK_ mice compared to non-Tg controls. Similarly, these species (*sn*-1 lysoPCs) either showed an upward trend or were significantly and similarly increased in old APP_WT_ mice (1.3- to 1.5-fold) (Fig. 2e). On the other hand, AA and DHA containing lysoPC species (*sn*-2 lysoPCs) were more extensively increased in APP_WT_ (by 1.7-fold) and in APP_OSK_ mice (by almost 4-fold) compared to non-Tg controls. The difference in the extent of increase in *sn*-1 versus *sn*-2 lysoPCs is consistent with two different processes responsible for their altered levels (PLA_2_ activity versus ROS-mediated oxidation, respectively).

Next, we proceeded to characterize the effects of APP_WT_ and APP_OSK_ overexpression on lysoPC content in middle-aged mice (12-month-old). Consistent with our NEFA data, total lysoPC content was not significantly altered within the brains of younger mice (Fig. 2f). Detailed analysis of lysoPC species revealed that APP_OSK_ tended to have higher levels of the same species that accumulated in old animals, although these increases were not statistically significant (Fig. 2f). Finally, it is important to mention that we also measured lysoPE content; however, no significant changes were detected between the total levels of the 3 analyzed mouse genotypes within middle-aged or old mice (data not shown). These results are consistent with previous reports claiming that cPLA_2_α is selective for AA-containing phosphatidylcholine (PC) [81].

### APP overexpression and oligomeric Aβ accumulation lead to dysregulation of cPLA_2_.

Our NEFA and lysoPC results strongly suggested that APP overexpression and oligomeric Aβ lead to increased PLA_2_ activity. Thus, we proceeded to characterize the major brain PLA_2_ isoforms by Western blot (WB). Specifically, we focused on characterizing cPLA_2_ (GIV) and iPLA_2_ (GVI), given that sPLA_2_ (GIIA) is absent in the APP-Tg and non-Tg mice used because of a frame-shift mutation in exon 3 in the C57BL6 strain [54].

**Fig. 3:**
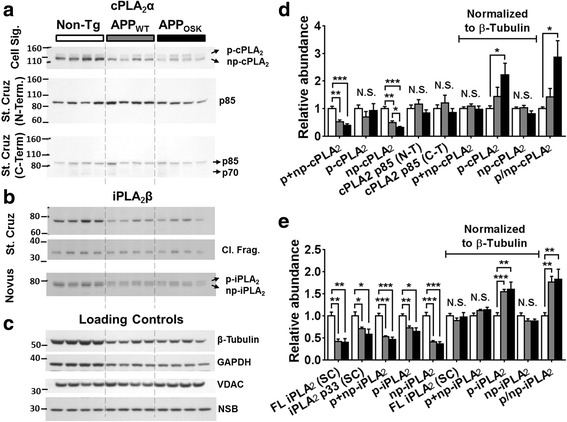
Effects of APP_WT_ and APP_OSK_ overexpression on the levels of the major phospholipase A_2_s in the brains of old mice. Cerebrum samples from non-Tg, APP_WT_ and APP_OSK_ were lyophilized, pulverized, and homogenized in NP40 buffer using a cooled bead beater. Total protein concentrations from NP40 supernatants were estimated by BCA protein assay. Representative Western blots using multiple antibodies against cPLA_2_ (**a**) and iPLA_2_ (**b**), as well as several commonly used loading controls (**c**). Relative intensities for cPLA2- (**d**) and iPLA2-related signals (**e**) were quantified using *ImageJ* software and normalized to total protein or β-Tubulin. The data represent means ± SE obtained from 4 animals/genotype. *p < 0.05, **p < 0.01, and ***p < 0.01. N.S. stands for not significant, p for phosphorylated, np for non-phosphorylated, FL for full length, SC for Santa Cruz, N−/C-T for N−/C-Terminus, and NSB for non-specific band

Intriguingly, even though the molecular weight of cPLA_2_α is 85 kDa, published SDS-PAGE-based WB studies have reported cPLA_2_α signals that range from 85 to 120 kDa [7, 20, 59]. We used 3 different cPLA2 antibodies purchased from 2 different companies (Cell Signaling and Santa Cruz) and observed 3 different bands (115 kDa, 120 kDa, and 85 kDa) depending on which antibody was used (Fig. 3a). The antibody from Cell Signaling recognized a previously described non-phosphorylated ~115 kDa cPLA_2_α band, which under physiological conditions appeared as the most intense band. An additional and previously described phosphorylated ~120 kDa cPLA_2_α band was also observed. Importantly, both of this bands were shown to be specific to cPLA_2_α since they were absent in cPLA_2_α KO mice. Longer exposure times revealed an additional ~85 kDa band (not shown). Interestingly, characterization of 2 different Santa Cruz antibodies (recognizing either cPLA_2_ N- or C-terminus) failed to detect the 115 and 120 kDa bands but effectively detected the 85 kDa band. Long exposures of the antibody against C-terminus cPLA_2_ (H-12, sc-376,636) also revealed a weak previously reported 70 kDa cPLA_2_ cleavage product band (Fig. 3a).

Unexpectedly, WB analysis of cPLA_2_α revealed a significant decrease of total cPLA_2_α levels (phospho + nonphosphorylated or 115 + 120 kDa bands) in both old APP_WT_ (reduced by 48%) and APP_OSK_ mice (reduced by 61%) compared to non-Tg controls (Fig. 3a and d) after normalizing to total protein or certain loading controls (like VDAC or COX IV). Surprisingly, several commonly used loading controls (like β-Tubulin, GAPDH and PCNA) were also significantly reduced in APP-Tg mice (Fig. 3c). These decreases paralleled the reductions observed in total cPLA_2_, in fact, if normalized against any of these common loading controls, total cPLA_2_ levels were not altered (Fig. 3d). Consistently with our lipidomics data, we observed a significant increase (2.9-fold) in the ratio of phospho- to non-phospho-cPLA_2_ in old APP_OSK_ mice and an upward trend (1.4-fold) in APP_WT_ mice compared to non-Tg controls (Fig. 3a and b). On the other hand no significant changes in the levels of the 85 kDa band were detected in any of the 3 antibodies used after normalizing to total protein or VDAC (Fig. 3a and d). The previously reported 70-kDa cPLA_2_ fragment [1, 5, 6, 30, 40, 119, 128] was very weak within all of the homogenates. The fact that this cleavage fragment did not accumulate in APP-Tg mice clarifies that the decrease in total cPLA_2_ is not a consequence of increased proteolytic cleavage.

### APP overexpression leads to dysregulation of iPLA_2_

Given that iPLA_2_ has been proposed to be highly specific to DHA cleavage/release [37, 69], to be enriched in AD-vulnerable brain regions (cortex and hippocampus) [87, 132], and to account for more than 70% of the brain PLA_2_ activity [132], we reasoned that the increases of free DHA found in our lipidomics analysis were likely due to the increased activity of iPLA_2_ in APP-Tg mice.

Surprisingly, as for cPLA_2_α, WB analysis revealed reduced levels of total iPLA_2_β (~78 kDa) in APP-Tg mice (60% reduction in both APP_WT_ and APP_OSK_) compared to non-Tg controls (Fig. 3b and e) after normalizing to total protein and/or certain loading controls (e.g., VDAC). Again, total iPLA_2_ levels paralleled the levels of several commonly used loading controls (e.g., β-Tubulin), so that if normalized against any of them, total iPLA_2_ levels were not altered (Fig. 3b and e). Importantly, our WB analyses were confirmed using two different iPLA_2_β antibodies (Novus and Santa Cruz) that target different epitopes (near the N- and C-terminus, respectively). Notably, one of the antibodies (N-terminus) yielded an additional band right above the band corresponding to full length (FL) iPLA_2_β (~80 kDa). It is reasonable to speculate that this additional band could represent phosphorylated iPLA_2_β. Importantly, the ratio of this putative phosphorylated iPLA_2_β to non-phosphorylated iPLA_2_β was significantly increased in APP-Tg mice compared to non-Tg controls (Fig. 3e). Consistently with the literature [136], we did find a ~ 33 kDa C-terminal fragment (when using the antibody against the C-terminal region of iPLA_2_β) (Fig. 3b and e). This cleaved iPLA_2_β fragment was significantly reduced in APP-Tg mice (by 30% and 42% in APP_WT_ and APP_OSK_, respectively). However, this decrease was mild compared to the more extensive reduction in FL iPLA_2_β. Consequentially, cleaved to FL ratios were significantly higher (50–70%, *p* < 0.05) in APP-Tg mice compared to non-Tg controls. These results suggest that under APP overexpressing conditions, FL iPLA_2_β is more likely to be processed than under physiological conditions. Nevertheless, increased FL iPLA_2_β cleavage ratios in APP-Tg mice do not fully explain the reduction of total iPLA_2_β (in which case the cleaved fragment would accumulate). Given that the reduction of total levels of iPLA_2_ paralleled the reduction seen for cPLA_2_, and “housekeeping” gene products (i.e., β-Tubulin, GAPDH, PCNA, and others), it seems likely that these decreases must be due to a more global effect (e.g., reduced expression of certain gene products or reduced density of certain cell types).

### DHA and AA accumulate in different and opposite brain regions

**Fig. 4:**
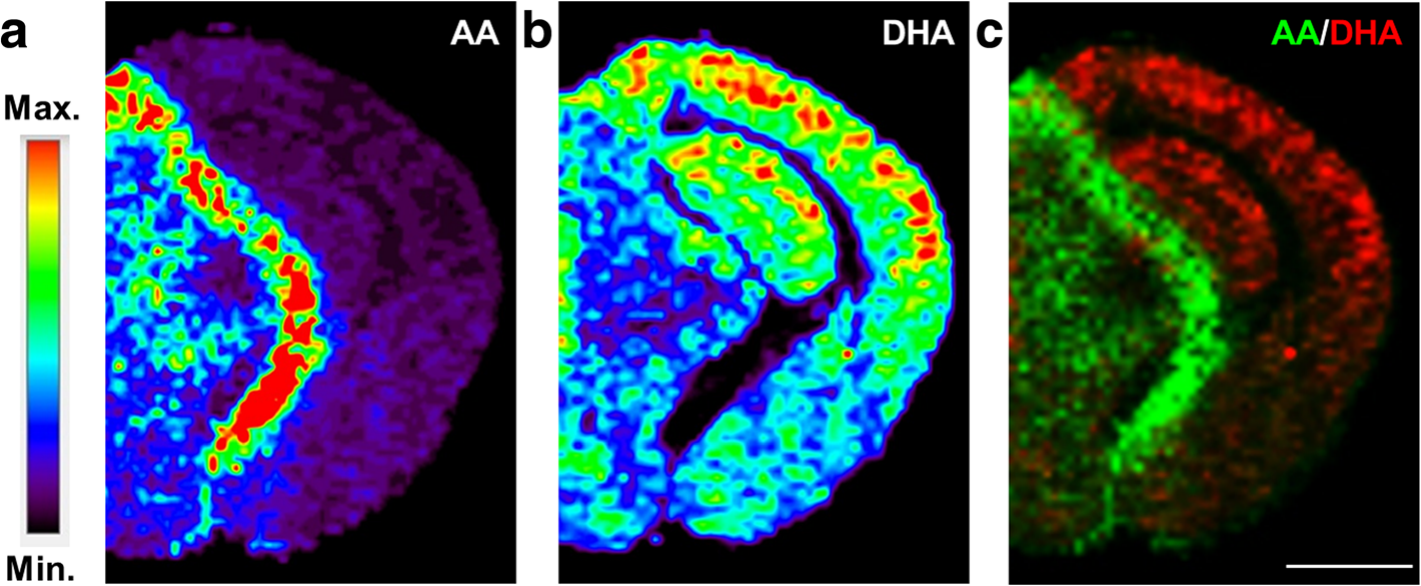
Distribution of arachidonic acid and docosahexaenoic acid within the brain. Non-Tg mouse brains were dissected, frozen, and sectioned coronally (10 μm sections). Representative MALDI-MS imaging heat maps from brain coronal sections (Bregma −2.4) for arachidonic acid (AA [M-H]^−^, *m/z* 303.23) (**a**) and docosahexaenoic acid (DHA [M-H]^−^, *m/z* 327.23) (**b**). **c** Merged image with AA in green and DHA in red, note their opposite distributions. MALDI-imaging resolution is 100 μm, scale bar = 2 mm

Given the opposite effects of AA and DHA (pro- and anti-inflammatory, respectively), we wondered whether their distribution in the brain would also differ under physiological conditions. Interestingly, MALDI-MS imaging analysis revealed that AA and DHA accumulated in different and opposite brain regions (Fig. 4). Imaging analysis of coronal sections revealed that AA signals were highest along the brachium of the superior colliculus and optic tract, while mild signals were seen within thalamic and hypothalamic regions, and low or virtually no signals were observed within the amygdala and cortical/hippocampal regions (Fig. 4a). On the other hand, DHA signals were highest within the cortex and hippocampus, while mild signals were seen within the amygdala, and low signals were detected within thalamic and hypothalamic regions (Fig. 4b). In summary, AA seems to concentrate along bundles of nerve fibers while DHA is most abundant within regions rich in pyramidal neurons. Thus, AA and DHA not only have opposing signaling effects, but they also have opposite distributions throughout the brain.

### Spatial distribution of cPLA_2_ and iPLA_2_ in the brain

**Fig. 5:**
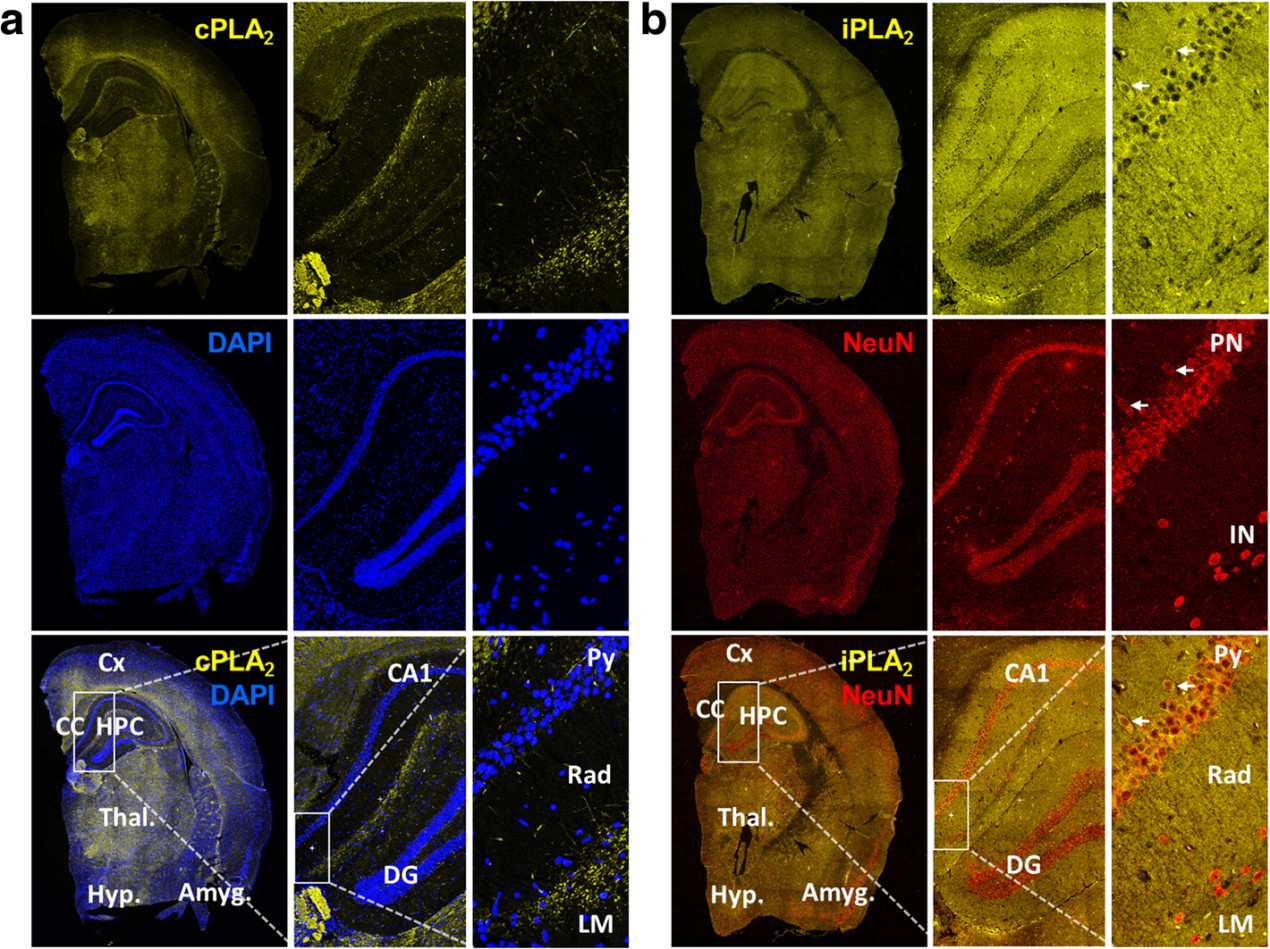
Distribution of cPLA_2_ and iPLA_2_ within the brain. Non-Tg mouse brains were dissected, fixed in 4% paraformaldehyde, cryoprotected, frozen, and sectioned coronally (8 μm sections). Representative immunofluorescence images were taken from brain coronal sections (Bregma −2) using phospho-cPLA_2_ (yellow) antibody and DAPI (blue) (**a**) or and iPLA_2_ (yellow) and NeuN (red) antibodies (**b**). The images show a whole hemibrain section using a 20× objective on a Nikon A1R VAAS inverted confocal (left panels in *A* and *B*). Cortex (Cx), corpus callosum (CC), hippocampus (HPC), thalamus (Thal.), hypothalamus (Hyp.), and amygdala (Amyg.). A zoom to hippocampal CA1/dentate gyrus (DG) (middle panels in **a** and **b**), and a zoom to the strata pyramidale (Py), radiatum (Rad) and lacunosum moleculare (LM) (right panels in **a** and **b**) were taken using a 40× objective. Note the clear axonal cPLA_2_ staining within myelin-rich regions and its absence within the cell bodies or dendritic arborization of pyramidal neurons (right panels, **a**). NeuN and iPLA_2_ co-localize within the perinucleus of pyramidal neurons (PN, white arrows), but not within the perinucleus of interneurons (IN). iPLA_2_ staining is also observed within pyramidal dendritic arborization (right panels, **b**)

We speculated that cPLA_2_ and iPLA_2_ should be expressed in different brain regions explaining the opposed distribution patterns observed for DHA and AA. Immunohistochemical analysis revealed that cPLA_2_ staining was most intense within regions rich in white matter tracts as well as within the thalamus and hypothalamus (Fig. 5a, left). In fact, cPLA_2_ staining highly resembled the typical staining of myelin-specific proteins. On the other hand iPLA_2_ staining was most intense within the hippocampus, while significant signals were also observed within thalamic, cortical, and amygdala regions (Fig. 5b, left). Contrary to cPLA_2_ staining, iPLA_2_ signals were virtually absent within white matter tracks (e.g., corpus callosum, CC). More detailed characterization at higher magnifications revealed that cPLA_2_ was enriched within the CC and at the border between hippocampal strata radiatum (Rad) and lacunosum moleculare (LM) (Fig. 5a, middle). cPLA_2_ signals were specific to myelinated axons and virtually absent in neuronal cell bodies (Fig. 5a, right). On the other hand, the strongest iPLA_2_ signal was found within neuronal cell bodies of the stratum pyramidale (Py) of the hippocampus. Interestingly, iPLA2 staining colocalized with NeuN (a pan neuronal marker) staining only within pyramidal neurons, and not within interneurons or granule cells of the dentate gyrus. In addition, iPLA_2_ was also observed within regions of dendritic arborization (e.g., Rad) (Fig. 5b, right). Similarly, iPLA_2_ and NeuN co-staining was also observed within pyramidal neurons in the cortex and amygdala (not shown at high magnification).

### Unraveling the kinases responsible for inducing iPLA_2_ phosphorylation in APP-Tg mouse brain tissue

To further confirm and better understand the mechanisms leading to cPLA_2_ and/or iPLA_2_ activation in APP-Tg mice, we proceeded to examine the kinases that have been demonstrated to phosphorylate cPLA_2_ as well as putative kinases that may phosphorylate iPLA_2_. Previous studies have shown that phosphorylation of cPLA_2_ *α* by mitogen-activated protein kinase (MAPK) (p42/44 and p38) at Ser505 and by Ca^2+^/calmodulin-dependent protein kinase II (CaMKII) at Ser515 stimulate its catalytic activity [34, 50, 60, 66]. Finally, it has also been shown that okadaic acid activates cPLA_2_ *α* and stimulates AA release via a p54 kinase [117].

**Fig. 6:**
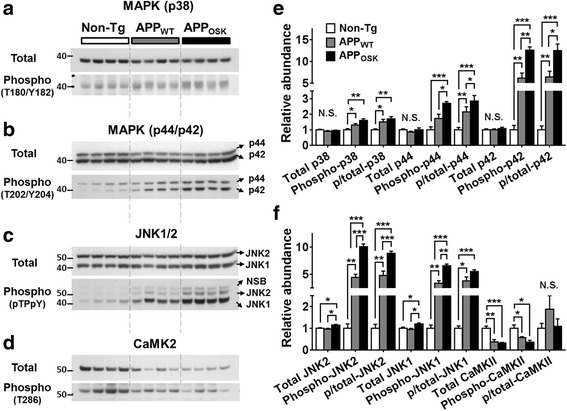
Effects of APP_WT_ and APP_OSK_ overexpression on the levels of known PLA_2_ kinases in the brains of old mice. Cerebrum samples from non-Tg, APP_WT_, and APP_OSK_ were lyophilized, pulverized, and homogenized in NP40 buffer using a cooled bead beater. Total protein concentrations from NP40 supernatants were estimated by BCA protein assay. Representative Western blots using antibodies against total and phospho-MAPK p38 (**a**), p44/p42 (**b**), JNK1/2 (**c**), and CaMK2 (**d**). **e-f** Relative intensities were quantified using *ImageJ*. The data represent means ± SE obtained from 4 animals/genotype. *p < 0.05, **p < 0.01, ***p < 0.01, and N.S. as for not significant

We decided to characterize the 3 classical MAPK families: p38, p44/p42 (also known as ERK1/2), and JNK1/2 since the first two have been shown to directly phosphorylate cPLA_2_ and the fact that JNK2 has a molecular weight of 54 kDa. MAPKs are catalytically inactive in their base form and activated by phosphorylation (within residues of their activation loops). Thus, WB analysis using both total and phospho-specific antibodies provides a straightforward means to estimate their relative activities under different conditions (calculated as phospho- to total MAPK ratios). As expected from our lipidomics and PLA_2_ WB analyses, we found that APP-Tg mice showed increased MAPK activities (i.e., higher phospho/total MAPK ratios) compared to non-Tg controls (Fig. 6a-c and e). These significant increases were most dramatic for p42, where phospho/total ratios were 6-fold higher in APP_WT_ and 13-fold higher in APP_OSK_ compared to non-Tg controls (Fig. 6b and e). Phospho/total JNK1/2 ratios were also extensively increased in APP-Tg mice (4 to 5-fold and 6 to 9-fold increases in APP_WT_ and APP_OSK_, respectively) (Fig. 6c and f). Similarly, phospho/total p44 ratios were 2- and 3-fold higher in APP_WT_ and APP_OSK_ (Fig. 6b and e); while the phospho/total p38 MAPK ratios were mildly but significantly increased in APP-Tg mice (by 50% and 70% in APP_WT_ and APP_OSK_, respectively) (Fig. 6a and e).

Next, we proceeded to measure the levels of phospho- to total CaMKII since it has also been shown to phosphorylate cPLA_2_ at a different residue (S515). However, we did not find any significant differences in phospho/total CaMKII ratios between APP-Tg and non-Tg mice (Fig. 6d and f), suggesting that activation of cPLA_2_ by phosphorylation in APP-Tg mice occurs via the MAPK, but not the CaMK2 pathway. It is important to mention that as for cPLA_2_ and iPLA_2_, the levels of total CaMKII were significantly reduced in aged APP-Tg mice compared to non-Tg controls. Again, this decrease paralleled those seen for typical “loading control” proteins. On the other hand, MAPKs were not significantly decreased in APP-Tg mice. Finally, analysis of middle-age mice revealed no significant differences in the levels of the analyzed phospho- or total kinases (data not shown), consistently with our lipidomics and PLA_2_ data.

### MAPK activation occurs independently of PKC activation in aged APP-Tg mice

**Fig. 7:**
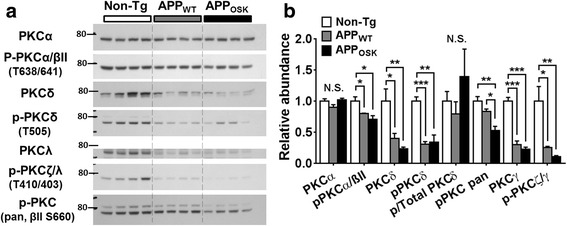
Effects of APP_WT_ and APP_OSK_ overexpression on the levels of total and phosphorylated PKCs in the brains of old mice. Cerebrum samples from non-Tg, APP_WT_, and APP_OSK_ were lyophilized, pulverized, and homogenized in NP40 buffer using a cooled bead beater. Total protein concentrations from NP40 supernatants were estimated by BCA protein assay. **a** Representative Western blots using antibodies against total and phospho-PKC isoforms. **b** Relative intensities were quantified using *ImageJ*. The data represent means ± SE obtained from 4 animals/genotype. *p < 0.05, **p < 0.01, ***p < 0.01, and N.S. as for not significant

We proceeded to assess whether MAPK activation occurred in a PKC-dependent manner in APP-Tg mice. For this purpose, we characterized multiple isoforms of conventional, novel, and atypical PKCs as well as different phosphorylation events (at the activation loop, turn motif, and hydrophobic motif of PKCs) that have been linked to increased PKC activity [55]. Total levels of conventional PKCα were not altered between APP-Tg and non-Tg mice, while the levels of phospho-PKCα/βII (T638/641) (autophosphorylation event at the turn motif) were actually slightly reduced in APP-Tg mice compared to non-Tg controls. Total levels of novel (δ) and atypical (ζ/λ) PKCs were extensively reduced in APP-Tg mice compared to non-Tg controls (Fig. 7a-b). Interestingly, the total levels of novel and atypical PKCs paralleled those observed for total PLA_2_s and commonly used loading controls. Similarly, phosphorylation levels of novel (δ) and atypical (ζ/λ) PKCs at the activation loop (T505 and T410/403, respectively) were also extensively reduced in APP-Tg mice compared to non-Tg controls (Fig. 7a-b). Therefore, phospho/total novel/atypical PKC ratios were not significantly altered between the 3 genotypes. Finally, we also characterized the final PKC autophosphorylation event at the hydrophobic motif which represents the third and last phosphorylation/activation step using phospho-PKC (pan) (βII S660) against all conventional and novel PKCs. Consistently with the results obtained from the other 2 phosphorylation sites analyzed, we also observed a significant reduction of phosphorylation levels at this position (Fig. 7a-b). Thus, after analyzing multiple PKC isoforms and phosphorylation events, we did not find any evidence of PKC activation in APP-Tg mice.

## Discussion

Seeking to better understand the role of fatty acid metabolism in AD and to unravel the mechanisms underlying its disruption we took advantage of the powerful technology of multidimensional mass spectrometry-based shotgun lipidomics (MDMS-SL) pioneered by our laboratory. At the same time, attempting to dissect if fatty acid dysregulation is linked to fibrillar and/or soluble Aβ accumulation, we took advantage of the APP_OSK_ mouse model where AD-like pathology and neurodegeneration occur as a consequence of high levels of soluble oligomeric Aβ in the absence of amyloid plaques. Importantly, besides non-Tg control mice, our studies also included APP_WT_ transgenic controls; this experimental design allowed us to distinguish between the effects of APP overexpression and those of oligomeric Aβ accumulation. To the best of our knowledge, this is the first study to demonstrate that (1) dysregulation of fatty acid metabolism in the context of AD occurs independently of amyloid plaques, (2) APP overexpression on its own induces an accumulation of unsaturated NEFAs (particularly AA, DHA, and OA) and lysoPCs while soluble oligomeric Aβ further exacerbates this accumulation of unsaturated FAs/lysoPCs, (3) cPLA2/AA and iPLA2/DHA accumulate in different and opposite brain regions, (4) AD-related fatty acid dysregulation is induced by PLA_2_ activation via MAPK-mediated phosphorylation in a PKC-independent manner.

### Fatty acid metabolism disruption in AD is mediated by increased PLA_2_ activity

Thanks to the fact that disruption of fatty acid metabolism results in specific signatures within the lipidome (depending on whether the alterations are induced through biosynthesis and/or degradation pathways), our lipidomics approach enabled us to not only demonstrate that both APP overexpression and high oligomeric Aβ content lead to significant and additive increases in unsaturated NEFAs within the brain of aged mice, but also to gain insights into the mechanisms underlying this disruption of FA metabolism. The lipidomics signature obtained strongly indicated that the accumulation of NEFA occurred as a consequence of increased FA cleavage. Specifically, analysis of the two major lysophospholipids classes revealed a dramatic accumulation of lysoPCs. Detailed characterization of specific lysoPC species indicated that FA cleavage in the context of AD seems to be induced by increased PLA_2_ activity as well as oxidative stress (revealed by an accumulation of 4-HNE and *sn*-2 lysoPCs). We validated this lipidomics-based hypothesis by analyzing the most abundant PLA_2_ enzymes in the brain by WB and provided evidence supporting a model in which increased PLA_2_ activity is mediated by phosphorylation of cPLA_2_α and iPLA_2_β.

These results are in agreement with multiple previous reports linking high AA content and cPLA_2_ activity/phosphorylation to AD (reviewed in [94]) [22, 95, 108, 109, 111, 113]. Furthermore, two separate groups have recently reported increased free DHA levels in human AD brains [82, 105]. In addition, the proportion of phospholipid-bound DHA has been reported to be decreased in AD brains [25], which is also consistent with increased FA cleavage rates. The fact that iPLA_2_ has been shown to preferentially cleave/release DHA from brain phospholipids [37, 69] together with our data from AD mice and human data from other labs showing increased levels of free DHA, strongly implicate iPLA_2_ as a potential driver of this accumulation. To the best of our knowledge we are the first ones to propose an association between iPLA_2_β and AD.

Taken together our results strongly suggest that both PLA_2_-mediated accumulation of free PUFAs and oxidative stress drive AD-related disruption of lipid metabolism. Furthermore, we think that oligomeric Aβ-induced NEFA accumulation might be associated with the “adipose inclusions” described by Alois Alzheimer more than a century ago.

### Opposite brain spatial distribution between cPLA_2_/AA and iPLA_2_/DHA

In vitro studies have shown that cerebral microvascular endothelium and astrocytes can produce DHA and AA [76, 77]; in contrast, neurons cannot produce PUFAs but get enriched with PUFAs if they are co-cultured with astrocytes and endothelial cells. Interestingly, MALDI-MS imaging analysis revealed that AA and DHA accumulate within different and opposite brain regions. We found that although free DHA is detected throughout the brain, it accumulates most strongly within cortical and hippocampal regions, both of which are rich in pyramidal neurons/dendritic spines and are severely affected in AD. Consistent with this observation, previous studies have reported that 50% of the weight of neuronal plasma membrane is composed by DHA [103, 121]. Our results suggest that even under physiological conditions, there is a high exchange between free and lipid-bound DHA, presumably due to the high levels of plasma membrane remodeling that occur within dendritic spines (which are particularly enriched in pyramidal neurons). On the other hand, AA levels were strongest along bundles of nerve fibers and moderate within thalamic and hypothalamic regions, while cortical and hippocampal regions showed negligible levels of AA. It is reasonable to speculate that the opposite localization of free AA and DHA within the brain could be evolutionarily related to their opposite roles as mediators of pro- and anti-inflammatory signaling pathways.

Given that epidemiological research has linked high DHA consumption with a lower risk of AD [79] and animal studies have reported a reduction of amyloid, tau, and neuritic pathology with oral intake of DHA [12, 38, 65], it could seem paradoxical that AD brains accumulate free DHA. However, it is important to consider that DHA consumption is likely to result in increased membrane-associated (lipid-bound) DHA content which is of structural and functional relevance; while phospholipid cleavage under pathological conditions is likely to result in reduced lipid-bound DHA and increased free DHA. In fact, in AD there is a dramatic loss of dendritic spines as well as a significant loss of neurons with a concomitant increase in the levels of astrocytes (reviewed in [58, 91]). This cell-type remodeling could explain the overall increase in free PUFAs reported here and by others [82, 105].

Supporting our proposed model in which iPLA_2_ activation induces free DHA accumulation, we observed a strong correlation between DHA MALDI-imaging maps and iPLA_2_ immunofluorescence. Specifically, we report iPLA_2_ immunolabeling within the perinuclear cytoplasm and dendritic arborization of pyramidal neurons. Importantly, these results are in agreement with a previous study that reported high iPLA_2_ expression within the hippocampus (i.e. in the nuclear envelope of neurons, dendrites, and axon terminals) and lower expressions within the thalamus and hypothalamus of monkey brains [87].

On the other hand, AA and cPLA_2_ histological studies also revealed an overlap in their localizations. Specifically, both AA and cPLA_2_ accumulated within nerve fiber bundles and showed significant levels within thalamic and hypothalamic regions, revealing that cPLA_2_ and AA release are highly specific to myelin-rich regions. Previous characterization of cPLA_2_ within the rat brain noted high activities and immunoreactivities in the hindbrain, with moderate and low activities/staining in the midbrain and forebrain, respectively [86]. These results are consistent with high cPLA_2_ levels/activities within myelin-rich regions. In fact, recent evidence has revealed a strong cPLA_2_ immunoreactivity within axons and oligodendrocytes [67], further supporting our data/model. Notably, myelin-rich regions besides having a high abundance of phospholipids/sphingolipids, are also highly surrounded by astrocyte processes (where cPLA_2_ expression has also been reported [135]). Taken together, AA release seems to occur preferentially within myelin-rich regions in a process involving oligodendrocyte, astrocyte, and/or axonal cPLA_2_.

### AD-related MAPK activation is independent of amyloid plaques

Our results revealed that all major MAPK pathways are activated by Aβ accumulation (both by APP_WT_ overexpression and even further by accumulation of mutant Aβ) in the absence of fibrillary amyloid deposits. These results are in agreement with previous reports demonstrating that all major MAPK pathways (i.e., ERK, JNK, and p38 pathways) are activated in vulnerable neurons in patients with AD (reviewed in [137]). Activated MAPK signaling pathways have been proposed to significantly contribute to AD pathogenesis through various mechanisms including regulation of APP, β- and γ-secretases, and induction of neuronal apoptosis (reviewed in [57]). Here we are adding cPLA_2_ (and potentially iPLA_2_ as well) activation to the list of mechanisms by which MAPK mediates AD pathologies. Furthermore, we demonstrated for the first time that soluble oligomeric Aβ is sufficient to dramatically activate MAPK pathways in the absence of amyloid deposition and that this Aβ-induced MAPK activation occurs in a PKC-independent manner.

### The importance of wild-type APP controls for mutant APP transgenic studies

Our results revealed that over time overexpression of WT APP is sufficient to induce significant alterations in a broad set of lipid and proteins classes. Although some of these effects were further exacerbated in APP mutant Tg mice comparted to APP_WT_ mice; we found several examples of markers that despite being altered between APP-Tg and non-Tg mice, were not significantly different between APP_WT_ and APP mutant mice (like some PKC isoforms, GAPDH, and β-tubulin). Our results clearly demonstrate that unless APP_WT_-Tg mice are included as controls, caution needs to be taken when concluding that a given effect is a consequence of a specific APP mutation(s) since such outcome could be partially or fully caused merely by transgenic APP gene expression.

Unfortunately, even though a plethora of mutant APP-Tg mouse models are currently available, only a handful of them have their respective APP_WT_-Tg mouse control available. In fact, the vast majority of published AD animal studies have based their conclusions on comparisons between mutant APP-Tg mice versus non-Tg controls. We urge the AD field to consider incorporating APP_WT_-Tg control mice into their experimental approaches whenever mutant APP-Tg mice are used. Alternatively, mutant APP-Tg results should be confirmed on human AD brain tissue and/or other non-Tg AD models, like the recently developed APP mutant knock-in mice [71]. Notably, our results also demonstrated that wild-type APP overexpression on its own is capable of modeling at least some aspects of AD (e.g., MAPK/cPLA_2_ activation and free PUFA accumulation). Consistently, overexpression of wild-type hAPP causes early onset familial AD in human carriers with APP duplications [73, 92] and presumably in Down’s syndrome [127].

## Conclusions

1. Soluble oligomeric Aβ-induced PLA_2_-mediated accumulation of free PUFAs leads to a disruption of lipid metabolism in AD independently of fibrillar amyloid.
2. cPLA_2_ activity leads to the release and accumulation of free AA within myelin-rich regions, while iPLA_2_ activity leads to the release and accumulation of free DHA within pyramidal neuron-rich regions.
3. Oligomeric Aβ is sufficient to dramatically activate MAPK pathways in the absence of amyloid plaques in a PKC-independent manner. This MAPK activation leads to increased PLA_2_ phosphorylation/activity.

## Abbreviations

4-HNE: 4-hydroxynonenal
AA: Arachidonic acid
AD: Alzheimer’s disease,
ADDL: Aβ derived diffusible ligand
apoE: apolipoprotein E
APP: Amyloid precursor protein
Aβ: Amyloid beta
CC: Corpus callosum
CIF: Calcium influx factor
COX: Cyclooxygenase
cPLA_2_: group IV Ca^2+^-dependent cytosolic
DHA: Docosahexaenoic acid
FA: Fatty acids
iPLA_2_: group VI Ca^2+^-independent PLA_2_
LM: Lacunosum moleculare
MALDI: Matrix-assisted laser desorption/ionization
MAPK: Mitogen-activated protein kinase
MDMS-SL: Multidimensional mass spectrometry-based shotgun lipidomics
MUFA: Monounsaturated fatty acid
NEFA: Nonesterified fatty acids
OA: oleic acid
Osk: Osaka
PKC: Protein kinase C
PLA_2_: Phospholipase A_2_
PUFA: Polyunsaturated fatty acid
Rad: Radiatum
sPLA_2_: group II secretory PLA_2_
Tg: Transgenic
